# Designing a Simple Fiducial Marker for Localization in Spatial Scenes Using Neural Networks

**DOI:** 10.3390/s21165407

**Published:** 2021-08-10

**Authors:** Milan Košťák, Antonín Slabý

**Affiliations:** Faculty of Informatics and Management, University of Hradec Králové, Rokitanského 62, 50003 Hradec Králové, Czech Republic; antonin.slaby@uhk.cz

**Keywords:** computer vision, fiducial marker, augmented reality, neural network, deep learning

## Abstract

The paper describes the process of designing a simple fiducial marker. The marker is meant for use in augmented reality applications. Unlike other systems, it does not encode any information, but it can be used for obtaining the position, rotation, relative size, and projective transformation. Also, the system works well with motion blur and is resistant to the marker’s imperfections, which could theoretically be drawn only by hand. Previous systems put constraints on colors that need to be used to form the marker. The proposed system works with any saturated color, leading to better blending with the surrounding environment. The marker’s final shape is a rectangular area of a solid color with three lines of a different color going from the center to three corners of the rectangle. Precise detection can be achieved using neural networks, given that the training set is very varied and well designed. A detailed literature review was performed, and no such system was found. Therefore, the proposed design is novel for localization in the spatial scene. The testing proved that the system works well both indoor and outdoor, and the detections are precise.

## 1. Introduction

Marker detection is a process of finding the area in an image with a unique character, sign, or color. They are used as visual cues that are easy to identify for computer vision systems. There are many ways and many existing systems that try to solve this problem. A broad spectrum of applications can use such markers. The primary examples include augmented reality (AR) applications, robot navigation, camera calibration, or pose determination in industrial environments.

**Figure 1:**
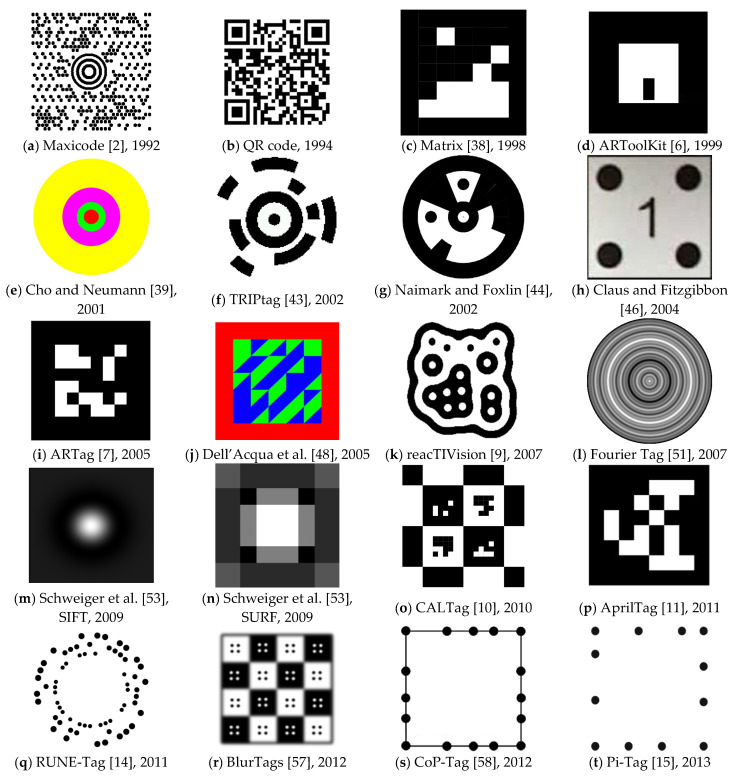
Examples of the most notable marker systems with their name (if available) and year of their publication.

Today, QR codes [1] (abbreviated from quick response codes) are the most typical and ubiquitous example of a marker system. They are usually used to carry textual information in the real world in a form that is easily and quickly readable by machines. Another example of such a system is Maxicode [2,3], which US Parcel Service uses. Both QR code and Maxicode are also ISO standards [1,4]. For examples, see Figure 1a,b.

QR codes [1] and Maxicode [3] are designed to carry precise and complex information in the spatial scene. Generally, AR applications do not require that. They only need a reliable way of localizing an object or localizing a camera in the real world. AR applications usually combine the real world with artificial elements, and marker systems help integrate those separate worlds by obtaining information about the surrounding natural environment.

There is a big group of marker systems that are designed for various purposes. Examples of marker-based systems include ARToolKit [5,6], ARTag [7], ARToolKit Plus [8], reacTIVision [9], CALTag [10], AprilTag [11,12,13], RUNE-Tag [14], Pi-Tag [15], CCTag [16], ChromaTag [17], and TopoTag [18]. For further examples and descriptions, see the related work section.

All mentioned marker-based systems share one common disadvantage. The kinds of markers they use have complicated shapes. A complicated marker shape is such a shape that contains too many significant features, which makes it hard to create. A key characteristic of a complex marker is its difficult reproduction by hand-drawing. However, there are reasons why the systems require using such shapes—they are usually used in situations where it is desirable to store more data in the scene (e.g., QR codes or ARToolKit). Generally, they want to provide more information about the item to which the marker is attached. For instance, they can provide a payment code [19], so the account number and the amount of money do not have to be manually typed. Yet, if the application requires only simple localization information, it is unnecessary to use complicated shapes.

Moreover, as shapes get complicated, it is always necessary to print them. A simple, well-designed marker can meet localization requirements by using only a necessarily small number of significant features, which then can be drawn only by hand if necessary. Such a simple marker can also be less obtrusive than classic markers. Generally, a simple marker shape should be possible to draw by hand if no printer is available.

The authors focus the research on the field of marker detection over a longer period. Their previous works include two implemented algorithms for single-color marker detection [20,21,22]. Even though the algorithms were functional, detection problems appeared in some cases. Due to the core principle of the detection algorithm, the marker detection was not sometimes precise enough. The algorithms work by dividing the image into subareas to make them computationally cheap. However, this leads to inaccurate detections if the marker happens to be divided into multiple subareas.

The goal of this work is to overcome the issues that previous algorithms have. Another goal is to develop a solution that allows the detection and localization of simple shapes, unlike other mentioned solutions of the QR code family, which require complicated shapes and forms that always need to be printed to achieve accurate detections. The new solution should be able to work with any marker color that is saturated enough. Simultaneously, it should be error-prone, so the user can also draw the marker by hand with a highlighter.

As the description of previous works of authors of this paper implies, designing an algorithm to meet all these requirements is challenging. However, a promising approach with artificial neural networks and deep learning might solve all the mentioned problems. Artificial neural networks find many usages in computer vision, so the defined problem should be solvable with them, too, as they should be capable of sufficient generalization to allow such detections.

This paper’s main contribution is designing a marker shape suitable for localization in the spatial scene. The marker needs to fit into four constraints that are defined. These constraints ensure that the necessary information can be obtained from the marker placed in the scene. Moreover, the shape needs to be simple, as already described. The paper then explores in detail different marker shapes and their advantages and disadvantages. A thorough investigation for each considered shape is performed, results for each shape are compared, and the process leads to the shape that has excellent detectability. The researched shape is carefully tested in different scenarios, including real-life situations, and the testing reveals that the shape is very usable, distinguishable, and simple.

## 2. Related Work

Markers represent a popular system for camera or object localization, and many publications on this topic can be found. The chapter first classifies the systems, and the following text chronologically focuses on 39 different marker systems that are the most related to this work. See Table 1 for a summary of the investigated systems.

Most of the systems do not focus on any specific environment or application area in which they are used. However, some authors have focused on less common environments and situations. For example, dos Santos Cesar et al. [23] evaluated several fiducial markers in underwater conditions. Bondy et al. [24] designed a marker system specifically for use in space operations.

Numerous comparative works have been composed. Zhang et al. [25] compared ARToolKit, IGD, SCR, and HOM systems. Shabalina et al. [26] compared ARTag, AprilTag, and CALTag. In 2020, Morar et al. prepared a comprehensive study that evaluated many systems in an indoor environment [27]. Some authors have theoretically focused on designing the best marker systems, e.g., [28,29].

On the other side of the spectrum, there are marker-less systems that extract information from the scene only, e.g., [30,31,32,33]. This process is usually more complicated and requires more computational time than when the marker is present. Several studies focused on comparing marker-based and marker-less systems have been conducted in [34,35,36,37].

Marker-based systems are advantageous in situations where recognition is needed with high precision, where natural features are not present in sufficient quality and quantity, and when it is convenient to place markers [29].

Marker systems can be divided by several characteristics:

- by the color of the marker
  ○ black/grayscale (e.g., ARToolKit, ARTag, Fourier Tag)
  ○ colored (e.g., ChromaTag, LFTag)
- by the shape of the marker
  ○ square (e.g., ARTag, AprilTag, CALTag)
  ○ circle (e.g., TRIPtag, RUNE-Tag)
  ○ other (e.g., ReacTIVision)
- by the primary target application
  ○ carry complex information (e.g., Maxicode, QR code)
  ○ carry ID-based information (e.g., ARTag, April Tag)
  ○ localization (e.g., SIFTtag, SURFtag)
  ○ camera calibration or camera pose estimation (e.g., CALTag, HArCo)

The purpose of this research is to find a suitable method of localization. However, as the systems usually can be used interchangeably in many areas, the chapter also summarizes other marker systems that provide precious inspiration when creating the new system.

Matrix [38] is a marker system published by Rekimoto in 1998. It is one of the oldest systems that use black square areas with smaller white squares inside that carry the information (see Figure 1c).

ARToolKit [6] was among the first marker systems targeting AR applications. It is still one of the most popular systems. The marker contains a square-shaped white area with a payload and a black border surrounding the white area (see Figure 1d). The system allows users to define their custom payloads and later detect the internal pattern by correlation against a database of predefined patterns. However, this leads to slow detection times when the database is extensive. The system was first presented in 2002 and inspired many future marker systems. Later designs surpassed ARToolKit’s performance. An improved version, ARToolKit Plus [8], was developed in 2005, and it uses binary-coded patterns instead of correlation.

Cho and Neumann [39] published an idea of a multiring marker system in 2001. It was among the first systems that used color markers. It was typical for a detection system to utilize thresholding segmentation of the image to find the black-and-white marker. This new system uses a multi-threshold segmentation. The shape of the marker consists of several concentric rings, each of a different color (see Figure 1e). A combination of different colors produces unique and distinguishable markers. The authors propose two different kinds of markers: constant width rings and proportional width rings. Figure 1e shows an example of a marker with proportional width rings.

Zhang et al. [25] did a comparative study of four different marker tracking systems—ARToolKit [6], Institut Graphische Datenverarbeitung (IGD) marker system, Siemens Corporate Research (SCR) marker system [40,41], and Hoffman marker system (HOM). The systems were chosen because all of them are used in AR applications and are freely available. The authors evaluated the systems for usability, efficiency, accuracy, and reliability. They conclude that ARToolKit has the fastest processing time on single images, while SCR is the fastest on video sequences. Marker recognizability is working similarly for all mentioned systems, but ARToolKit has the best results in small regions. Although this study’s outcomes are valuable, it needs to be noted that it was published in 2002, and therefore, it does not contain the most recent works. Appel and Navab used the HOM system for 3D reconstruction and documentation in an AR application [42].

Target Recognition using Image Processing (TRIPtag) [43] is a marker system published by López de Ipiña et al. in 2002. The tag consists of a bullseye structure (black central dot and a solid black ring around it) and two black concentric outer rings divided into 16 sectors on a white background (see Figure 1f). The system uses adaptive thresholding for the detection process and focuses on low-cost cameras found in mobile phones and PCs. The system allows the encoding of $3^{9}\;\left(=19,683\right)$ unique IDs, and it does not work well with occlusion.

Naimark and Foxlin [44] published a marker system used mainly for motion tracking in 2002. The shape of the marker consists of a circular shape of black and white areas (see Figure 1g). The authors focused on handling different contrasts in real-world images. The system requires at least three visible markers. Once a set of markers has been located, the system switches to tracking mode and checks only windows around previously located markers. This method allows for a significant acceleration of the detection process. The system was also submitted as a US patent [45].

Claus and Fitzgibbon [46] published a marker system in 2004, which addresses detecting markers in real-world natural scenes. The marker has a square shape with a white background with four black circles in each corner, and it is identified by the code placed in the middle (see Figure 1h). The system uses a cascade of classifiers, and it is among the first to apply machine learning for marker detection. They show how robustness to lighting and scale can be addressed with the machine learning approach. The accuracy of the trained detector is over 95% in various indoor and outdoor scenes. The same authors did a follow-up work [47] in 2005, focusing on the automatic calibration of a position tracking system.

ARTag [7] is a marker system developed by Fiala in 2005. The shape of the marker is inspired by ARToolKit [6]. However, instead of letting users choose possible payloads, ARTag comes bundled with predefined markers, each corresponding to a unique ID. The system has a total of 2002 available markers. The marker consists of a black border, and there are black squares on a white background in the middle of it (see Figure 1i). The system detects markers from edges, and it also introduces error corrections. In 2010, Fiala also wrote an article [29] summarizing ways to design “highly reliable fiducial markers”.

Dell’Acqua et al. [48] presented a color tag detection system in 2005. It is one of the first systems that use squared colored markers. The authors take advantage of color markers, which makes them easily detectable in complex scenes. It also leads to better performance in poor illumination conditions. The proposed tag consists of a 5 × 5 grid of square blocks, which are divided diagonally. That leads to having 50 triangles, each having green or blue color (see Figure 1j). The system was inspired by Kawano et al. [49], whose system shares the same ideas but works with black-and-white markers.

ReacTIVision [9] is a blob detection system developed by Kaltenbrunner and Bencina in 2007. Unlike previous systems that use a rectangular-shaped marker, this framework is based on amoeba markers (see Figure 1k). The amoeba geometry was obtained by a genetic algorithm. The position of the amoeba symbol is the centroid of all found nodes. The framework is free and open-sourced [50].

Fourier Tag [51] is a fiducial marker system developed by Sattar et al. in 2007. The system is based on a frequency analysis of the image to decode the patterns and gains its name after the Fourier transform used for processing. The authors argue that systems like ARTag [7] do not provide satisfactory results when the viewing conditions deteriorate, for instance, with camera distance or camera noise. Fourier Tag is addressing these issues of low resolution. The marker consists of circles whose diameter increases with distance from the center (see Figure 1l). Xu and Dudek [52] did a follow-up implementation in 2011. They focused on configurable payload capacity and added the possibility of rotation detection.

Schweiger et al. [53] designed specific markers that trigger large responses with SIFT [30] and SURF [54] methods that are usually used in marker-less detection systems and can detect features in a scene without any markers. With this approach, the authors focused only on optimal marker shape design without worrying about designing the detection system. However, the system can provide only one marker for the SIFT method (see Figure 1m) and two markers for the SURF method (see Figure 1n). Nevertheless, that is not an issue, as the authors’ “goal is to have a highly detectable low-cost marker, not necessarily a uniquely detectable one” [53]. The work was published in 2009.

CALTag [10] is a marker pattern developed by Atcheson et al. in 2010. It is specifically designed for camera calibration. The marker area is like a checkerboard (see Figure 1), where every corner is used as a calibration point. With this approach, they achieve accurate calibration even with a significant degree of occlusion. The authors also explain why using ARTag [7] is not ideal for camera calibration.

AprilTag [11] is a marker system developed by Edwin Olson and published in 2011. The marker patterns are similar to those of ARToolKit [6] and ARTag [7] systems (see Figure 1p). However, this new system is more robust to lighting variations and occlusions, and it can encode 4164 unique codes, which is twice more than ARTag. ARToolKit cannot handle almost any occlusions, and ARTag can only work with small partial occlusions. AprilTag incorporates a fast and robust line detection system and a more reliable digital coding system and aims for more robustness to warping and lens distortion. In 2016, Wang and Olson [12] redesigned the detector and improved robustness and efficiency. The new detector is around 1.5 times faster, while the tag coding remained unchanged. AprilTag is free and open-sourced [55].

RUNE-Tag [14] is a marker system that was published by Bergamasco et al. in 2011. The system was developed with an emphasis on strong occlusion resilience. Unlike the previous systems, this one is formed by dots placed in circles (see Figure 1q). The RUNE-129 version achieves a 100% recognition rate with 50% occlusion and a 67% recognition rate with 70% occlusion. The detection precision comes at a cost. The authors admit that their system has a lower detection speed than other systems, being around a magnitude slower than ARToolKit Plus, but still running in real-time. Also, the system’s accuracy degrades with higher viewing angles. The authors further improved their system in 2016 [56]. Pi-Tag is a system of a slightly different design by the same authors [15].

BlurTags [57] is a marker system published by Reuter et al. in 2012. The system is focused on the detection of a blurred pattern. The marker is designed as a checkerboard containing black and white squares that are slightly blurred. Dots in each square with the opposite color are the payload (see Figure 1r). With this design, the cameras can be calibrated with different focus settings. The authors compare their design with the CALTag system [10], and they state that the new system has better resistance to higher levels of blur.

Connected Points Tag (CoP-Tag) [58] is a marker system developed by Li et al. in 2012. The marker consists of a square frame with exactly 16 dots, where each side has five dots (see Figure 1s). The system can detect a marker with up to 62.5% occluded dots, which means that only six dots must be visible. The authors also show that the system works well with noise and blur.

Pi-Tag [15] is a marker system published by Bergamasco et al. 2013. It is a follow-up work on RUNE-Tag [14], which was developed by the same team. The design of Pi-Tag is similar to CoP-Tag [58]. It consists of 12 dots placed on an imaginative square frame. Each side has four dots (see Figure 1t). The detection requires between 10 and 150 ms, being an order of magnitude slower than ARToolKit Plus [8]. The system can deal with moderate occlusion, giving good results when almost half of the dots are not visible.

**Figure 2:**
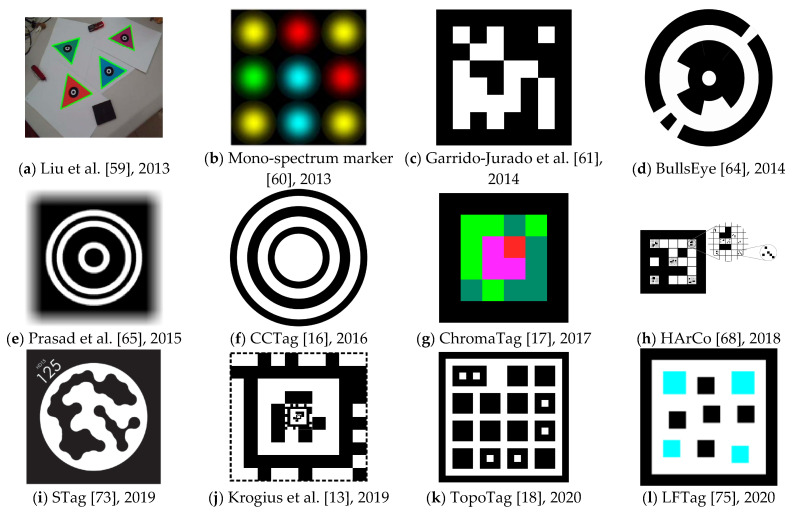
Examples of the most notable marker systems with their name (if available) and year of their publication.

Liu et al. [59] published a marker system that uses color markers (see Figure 2a) in 2013. High contrast between black and white in other systems makes the marker easily extracted. However, the adaptability to illumination changes is low. Using a color marker should overcome these issues. Also, a color marker usually matches the surroundings more naturally. The system’s primary goal is tracking the target object in real-time and registering a virtual 3D object. The authors state that RGB and HSV color models are not flexible enough for illumination changes. Therefore, they present a new method for recognizing a color marker in an image. The authors do not use a quadrilateral shape, as many other authors do, but instead they use an equilateral triangle. The main drawback is that the authors only compare their solution with ARToolKit [6], which was already 11 years old and overcome by more recent advances. The newly proposed method is also slightly slower than ARToolKit’s method.

Mono-spectrum marker [60] is a marker system published by Toyoura et al. in 2013. The marker design is focused on the ability to detect markers in blurred or defocused images. According to the authors, such situations often occur with camera motion or with fixed camera focal distance. The approach has a high computational cost, and the authors recommend using GPUs. However, this should not be an issue anymore at this time. See Figure 2b for a marker example.

Garrido-Jurado et al. [61] designed a marker-based system that aims for AR applications and robot localization. The system is based on square markers (see Figure 2c). The authors concentrate on the automatic generation of markers with an arbitrary size of the marker and the size of the set. Their most significant advantage is generating a marker dictionary according to the current situation’s needs. Previous solutions have used fixed-sized dictionaries. Using a variable-sized dictionary has some benefits. If the number of required markers is small, it is more reliable to use a small dictionary, reducing the inter-marker confusion rate. On the other hand, when an application requires a higher number of markers, it is still possible to create such a dictionary while keeping in mind that the error rate might be slightly higher. The work has been implemented into the ArUco library [62]. The paper was published in 2014. In 2016, the same authors further investigated the possibilities of generating marker dictionaries with mixed-integer linear programming [63]. That outcome was also added to the ArUco library.

BullsEye [64] is a marker system published by Klokmose et al. in 2014. It is specifically designed and optimized for processing on GPU. The authors aim for applications in table-based interaction and, therefore, the detection system must be very accurate. The authors state that the system provides sub-pixel accuracy down to a tenth of a pixel. The system can find the center coordinates, find the rotation angle, and extract the unique identifier. The performance of the marker is real-time. The authors mainly compare their work with the reacTIVision [9] system. The marker consists of a central white dot surrounded by a black ring. Partial black rings further surround this ring (see Figure 2d).

Prasad et al. [65] published a marker detection system in 2015. The system aims at drones and quadcopters that need information about their surroundings. However, these devices are subject to quick and unstable motions, and images from their cameras usually suffer from significant motion blur. The proposed design uses concentric circles (see Figure 2e) that are easier detectable with severe motion blur in the image. At the time of the publication, the system could not run in real-time, needing around 0.3 s to process a frame.

CCTag [16] is a marker system developed by Calvet et al. in 2016. It is focused on enhancing blur robustness. The shape of the marker consists of black rings on a white background (see Figure 2f). The thickness of the rings is used to encode information. The experiments showed that the detection system works well with high levels of occlusion and motion blur. However, the detection is relatively slow, reaching four frames per second with i5-4590 and 11 frames per second with GTX 980 Ti on an image with 1280 × 720 resolution. The system is open-sourced [66]. Before CCTag, in 2012, the same authors developed a system C^2^Tag [67], on which CCTag is based.

ChromaTag [17] is a marker system published by DeGol et al. in 2017. The authors propose color gradients to speed up the detection process. The system is 92 times faster than CCTag [16], 22 times faster than RUNE-Tag [14], and 16 times faster than AprilTag [12]. Using Intel i7 3rd generation, the system achieves an average of 926 frames per second (FPS), making it one of the fastest systems available. It achieves 709 FPS for true positive detections and 2616 FPS for false negatives (because all images in the test set contained a marker). Such speed is essential as marker systems are usually used in systems that do other heavy computations on top of the detection process. The system achieves similar or better detection accuracy than the mentioned systems. However, the authors admit that the detection fails at long distances. They recommend AprilTag as a better option for these conditions. See Figure 2g for an example of a ChromaTag marker.

HArCo [68] is a marker system published by Wang et al. in 2018. The authors focus on a hierarchical design that would allow the nesting of multiple markers. Similar solutions were proposed by Tateno et al. [69] in 2007 and in a system called Fractal Markers [70] in 2019. However, these systems are always nesting one marker inside another single marker. HArCo allows multiple markers nesting into a single marker (see Figure 2h). The average processing time of the system is four milliseconds, which allows real-time performance.

Susan et al. [71] investigated the possibility of using the Kullback-Leibler (KL) divergence measure [72] for marker detection. The authors compare their work with ARToolKit [6], which allows users to set custom markers and later detect them using correlation with the markers’ predefined database. Susan et al. experimented with replacing correlation by KL divergence for calculating similarities with the predefined database of markers. In such a case, KL divergence can act as the distance between two probability distributions defined by processed markers’ pixel intensities. The authors state that the new technique provides better detection performance than other methods. The paper was published in 2018.

STag [73] is a marker detection system proposed by Benligiray et al. in 2019. The focus of the system is for stable and not jittered detection. Therefore, the marker contains both a squared frame and a white circle inside. Combining them provides the advantages of both shapes, and STag is one of the first systems to utilize such a marker. The payload consists of black dots inside the white circle. These dots are repeatedly morphologically dilated and eroded. Filling the gaps between the dots allows correct reading in the case of slight localization errors. It also results in fewer edge detections. Moreover, the detection works well with occlusion. See Figure 2i for an example of a marker. STag library size is variable and ranges between 6 and 22,309 possible markers. The authors compare STag with ARToolKit Plus [8], Garrido-Jurado et al. [61], and RUNE-Tag [14]. Out of these systems, STag has superior detection accuracy while being slightly slower than ARToolKit Plus and Garrido-Jurado et al., but still running in real-time. The system is free and open-sourced [74].

Krogius et al. [13] designed a marker system that focuses on easy customization for each application. The marker can have high data density, have a circular shape, or be recursive (see Figure 2j). The work has been incorporated into the AprilTag system [11,12] and is referred to as AprilTag 3, sharing many similarities with previous versions of the system.

TopoTag [18] is a marker system published by Yu et al. in 2020. The shape of the marker is highly customizable. The basic shape consists of a black frame with black squares on a white background, and some of these squares contain a smaller white square. However, the black frame can be of any shape (authors give an example of a butterfly’s contour). The inner squares can also be of any shape, and the authors give examples of a circle and a hexagon. See Figure 2k for an example of the basic shape. The dictionary size is variable, and the authors state that its generation is much faster than in AprilTag [11] and ArUco [61]. The generation of similar size dictionaries takes 4.1 s in TopoTag, minutes for ArUco, and days for AprilTag. The detection performance is real-time and similar to ARToolKit [6], AprilTag 1 and 2 [11,12], and ChromaTag [17]. The detection precision is slightly better. The tests were performed on 169,713 real-world images. The detection system works well with up to 10% occlusion.

LFTag [75] is a marker system developed by Ben Wang and published in 2020. It is designed to resolve rotational ambiguity and to be localized even in challenging lighting and perspective conditions. Compared to AprilTag 3 [13] and TopoTag [18], LFTag offers a bigger dictionary size, which can be virtually unlimited (over 100 bits). The marker’s main limitations are occlusion and bending. The detection performance is real-time, comparable to AprilTag 3 and TopoTag. The marker always contains a black frame with a black or color square on a white background inside (see Figure 2l). The system is free and open-sourced [76].

### Summary

Most of the mentioned systems are like today’s ubiquitous QR codes, whose goal is to store complex machine-readable information in the spatial scene. This work aims to design a system suitable for augmented reality applications that do not require information placement in the scene. For such applications, the marker must be easily detectable and possible to acquire its position, rotation, size, and perspective skew, which are necessary for replacing the marker with a virtual object later. Only a minority of previous systems investigate a similar solution, for example, SIFTtag and SURFtag [53]. However, these two tags have rotational ambiguity. Only a few marker systems support rotation detection, namely Fourier Tags [52], BullsEye [64], and LFTag [75]. Some systems are focused on detection in blurred images, namely BlurTags [57], CoP-Tag [58], Mono-spectrum marker [60], Prasad et al. [65], and CCTag [16].

The new proposed marker system works well with blurred images and does not require a precisely printed marker. The new marker has rotation unambiguity, and it is usable for detecting position, rotation, size, and perspective projection. Testing with partial occlusion of the marker was not performed.

A characteristic feature of all previously mentioned systems is that they require a complex shape that needs to be printed. However, a system that supports an easy marker should not require this. Our proposed system works even with many imperfections that arise when not printing the marker but drawing it by hand.

The author’s research has been focused on marker detection for some time now [20,21]. Previously implemented solutions were focused on detecting single-color markers in an image. Both algorithms did not achieve the expected results.

The first one [20] describes a GPU algorithm that divides the image into rectangular sub-areas of the same size in which a marker is localized. The main problems had appeared when the marker was split between two or more regions, and consequently, its detection might be less reliable. Such situations did not often occur in the test environment, but it was inappropriate to ignore the problem. Also, the system was able to detect only the position.

The second version [21] of the algorithm eliminated this problem by the horizontal and vertical projection of the segmented area of the color marker. The image pixels are scanned in all columns and rows. Then, the pixels with the marker color are summed. Afterward, the coordinate at which the center of the marker is the most likely to be located is obtained using the weighted arithmetic mean.

Both algorithms are parallelized and were implemented using GPU shaders. One of the advantages of those prototype applications was that they were implemented in a WebGL environment, making them available across all major platforms.

## 3. Materials and Methods

### 3.1. Methodology

The proposed evaluation methodology is based on six parameters—IoU (intersection-over-union) value, false positive count, false negative count, percentage of false negative cases, precision, and recall.

**Figure 3:**
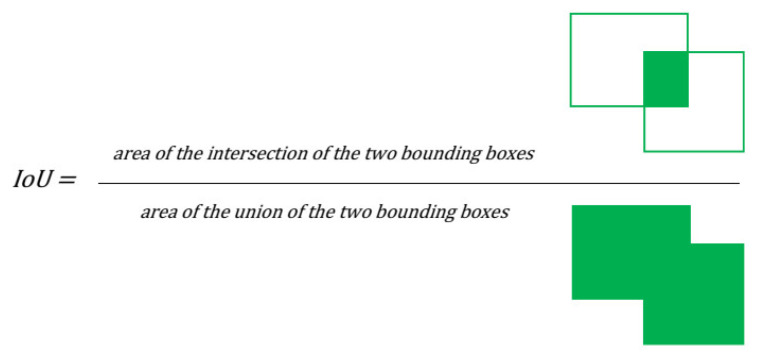
Graphical representation of intersection over union metric.

The IoU value (also known as the “Jaccard index”) is calculated as the ratio of the area of the intersection of the detected and ground truth bounding boxes and the area of their union, hence the name intersection-over-union (see Figure 3). The result is always in the range 〈0;1〉, where a zero value means a bad result and a false positive detection. A value of one means perfect detection with a deviation of fewer than 0.5 pixels in both dimensions. The final IoU value for multiple images is calculated as an arithmetic mean of all IoUs of the individual images in the test dataset. If there are more true positive detected bounding boxes for one ground truth bounding box, then the one with the highest IoU value is used to calculate the arithmetic mean.

The false positive case is when a bounding box detected in the image, but no marker is present in that position. Furthermore, all cases with IoU lower than 0.2 are also counted as false positives as these detections are poor and cannot meet previously defined criteria for ideal marker shape.

The false negative case is a marker that was not detected by any bounding box. All test dataset bounding boxes that were not detected are counted.

For a better understanding of the metrics, a summary of all four categories and their theoretical count are presented:

- true positive cases (TP)—all markers that were correctly detected;
- false positive cases (FP)—all *detected* markers that do not correspond to any *true* marker;
- true negative cases (TN)—any detection algorithm can detect a virtually unlimited number of possible bounding boxes even in a single image; therefore, all places in an image that do not have any detected marker while there is no true marker are considered as true negatives; there can be an unlimited number of such bounding boxes, and we consider true negative count always zero—this limits the usage of some statistical metrics (e.g., accuracy, which would always be 1);
- false negative cases (FN)—all *true* markers that were *not detected*.

The percentage of false negative cases gives a quick look at how many markers are missed by the detection algorithm. It is obtained simply by dividing the number of false negative cases by the total number of true markers in all images.

The percentage of false positive cases is not considered because this number does not make sense in the case of detection. Marker can be possibly detected anywhere in the image, and therefore theoretically, an infinity of false positive cases can be obtained if the detection is misbehaving. Thus, it does not make sense to divide this number by the total number of markers.

Precision is a number that describes how many of the detected bounding boxes are relevant. Recall tells how many of the relevant bounding boxes are detected. In our case, the true positive number is not directly available but is calculated by subtracting the false negative count from the total number of markers:

$$precision=\frac{TP}{TP+FP}\;recall=\frac{TP}{TP+FN}$$

$$recall=\frac{TP}{TP+FN}\;recall=\frac{TP}{TP+FN}$$

### 3.2. Theoretical Design

As it was explained, all the current systems require complicated shapes. This work aims to design a marker system that utilizes a much simpler shape that can be drawn by hand or be printed. That means that the system must be able to work with many imperfections that arise by handmade drawing. The goal of this marker system is to allow localization. Therefore, a simple shape should be sufficient when compared to shapes that carry more information. To provide all necessary information as requested by augmented reality applications, the marker’s shape needs to meet all the specified criteria. These criteria are:

- localization of the marker (preferably its center);
- detection of rotation angle in 360° relative to the viewer;
- detection of the whole marker to obtain its relative size;
- and having a shape that supports obtaining of perspective transformation.

Many tests with different shapes have been conducted to find the shape that meets all the mentioned conditions while keeping false positive and false negative detection counts low.

### 3.3. Dataset

Several different datasets were used during the tests. Artificially generated markers of a given marker shape were used to speed up finding the ideal shape. These generated markers were used in the first tests to avoid creating natural scenes with many different marker shapes that were considered. Markers were generated into real-world images. The first version of the dataset used around 3000 images and the dataset was continuously extended through the tests to a final set of around 9000 images. Every version of the dataset was consistently divided in the ratio of 7 to 2 to 1 to the train, validation, and test subsets.

The first datasets contained predominantly exterior images, and a dedicated dataset with interior images was created. It consisted of 2600 images that were found using these keywords: “bedroom”, “kitchen”, “living room”, “office”, and “home office”. In the end, a combined exterior and interior dataset with around 11,000 images was created.

The third dataset with real markers in natural scenes was created only with the final marker shape. It consisted of 550 images.

### 3.4. Neural Network Architecture

The YOLOv3 [77] neural network architecture was used for the implementation. It is one of the YOLO detectors that are state-of-the-art detectors using neural networks. This family of detectors is used for many detection tasks, for example, faces [78], tomatoes [79], forest fires [80], or traffic signs [81]. It requires only images and a corresponding file with bounding box information. Before entering the neural network, the images are resized to 416 × 416 pixels resolution. The training was carried out on a laptop with NVIDIA GeForce GTX 1060 Mobile graphics card. The same machine was used for performance testing.

## 4. Results

Ten iterations of tests were designed with different marker shapes to find the best marker design that would match the defined criteria. Some of the tested marker shapes did not meet all the specified requirements for a marker shape. However, it was crucial to perform thorough tests of various marker shapes to see which kinds have good results. The following iterations used this knowledge to design the final marker shape that has good testing results and meets the criteria simultaneously.

After the final marker shape is established, a follow-up algorithm is presented. The algorithm obtains the necessary information from the detected area, which contains the marker. This information is the output of the solution and is meant for usage by augmented reality applications.

### 4.1. Initial Test Iterations

The testing started with eight shapes (see Table 2). None of these shapes met all the criteria, but the shapes were chosen as a starting point in the first iteration.

- filled rectangle;
- empty rectangle;
- filled triangle;
- empty triangle;
- star of thickness 2;
- star of thickness 1;
- star of thickness 1 surrounded by a rectangle;
- and @ symbol.

**Figure 4:**
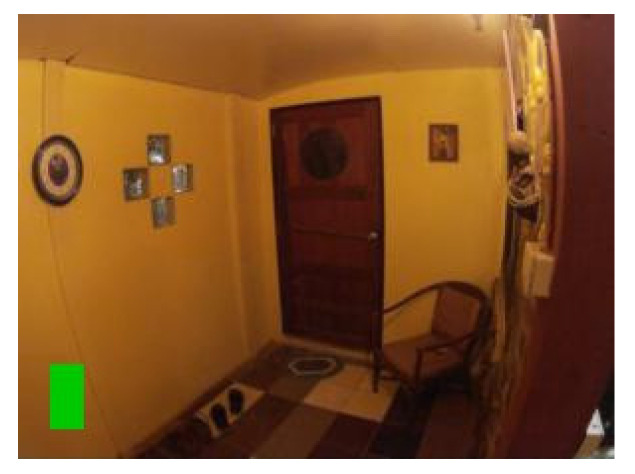
“Filled rectangle”.

The shape of the “filled rectangle” (see Figure 4) had a high count of false positive cases. That may be because there are many one-colored areas of rectangular shapes in the tested scenes. Also, this shape was not suitable because it did not meet the criteria for rotation detection. Although, it was used as the basis for the follow-up shapes in the later iterations.

**Figure 5:**
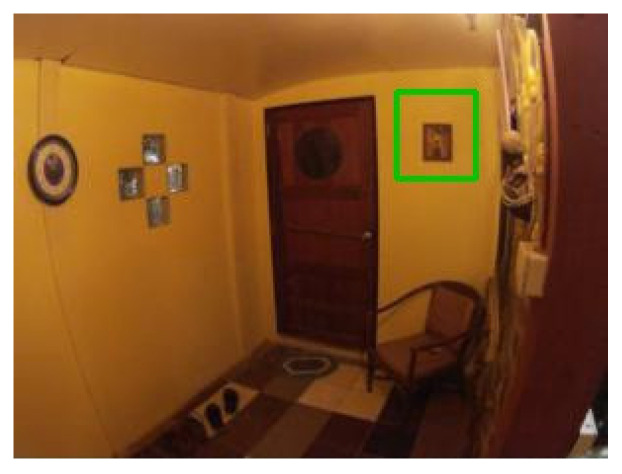
“Empty rectangle”.

The shape of the “empty rectangle” (see Figure 5) was similar to the “filled rectangle” having a high count of false positive cases. Furthermore, it was also not suitable because it did not meet some of the described criteria.

**Figure 6:**
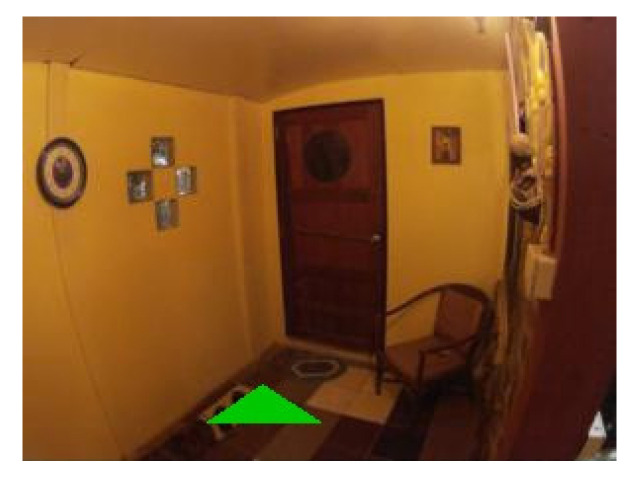
“Filled triangle”.

The shape of the “filled triangle” (see Figure 6) was an attempt in another direction. It was thought that such a shape might be better detectable. This idea was correct as the shape had low counts of false positive and false negative cases and a high IoU value. However, the shape met only one of four criteria, and without any potential to fix the issues, it was also rejected.

**Figure 7:**
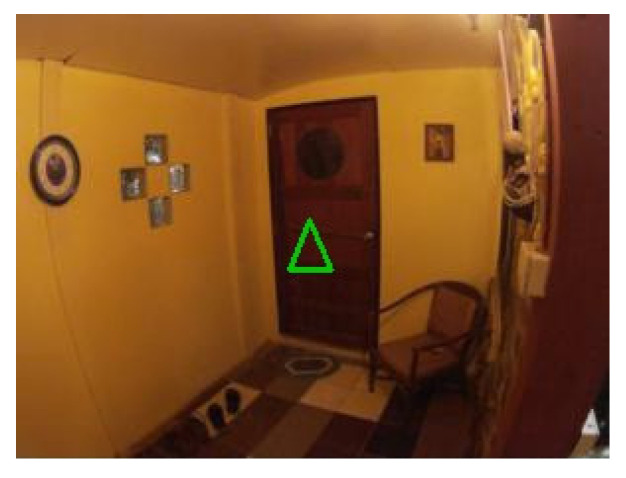
“Empty triangle”.

The results of the shape “empty triangle” (see Figure 7) were very similar to “filled triangle”, and with the same conclusions, it was also dismissed.

**Figure 8:**
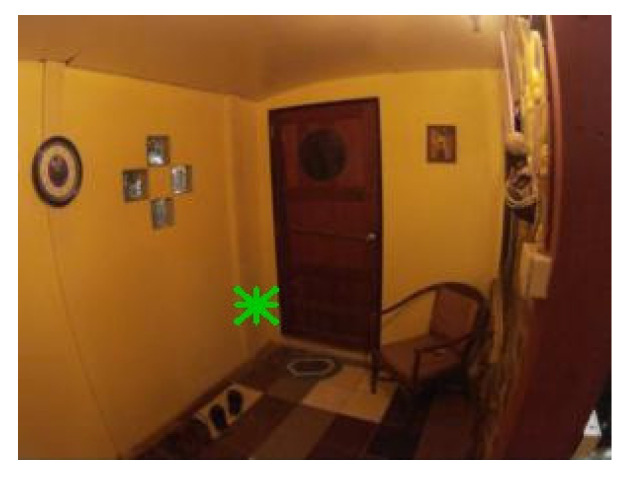
“Star of thickness 2”.

**Figure 9:**
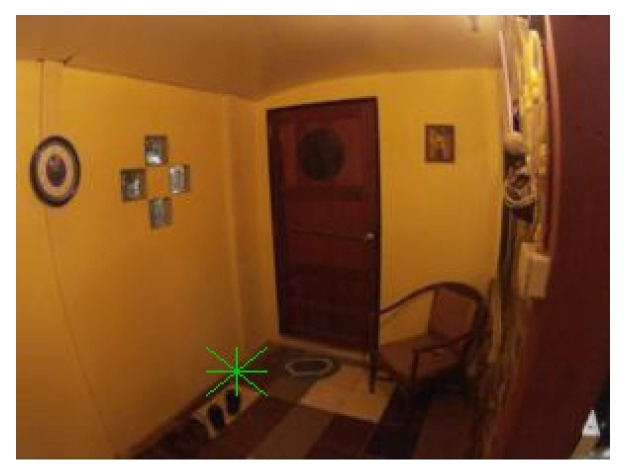
“Star of thickness 1”.

The shapes of the “star of thickness 2” (see Figure 8) and the “star of thickness 1” (see Figure 9) were tested to see if shapes with clearly defined center without a clearly defined body might lead to good results. Especially the thicker one was very nicely detectable with low counts of false positive and false negative cases. However, both shapes did not meet three out of the four criteria. Nevertheless, the idea of having a distinguishable center was used when designing the follow-up shapes.

**Figure 10:**
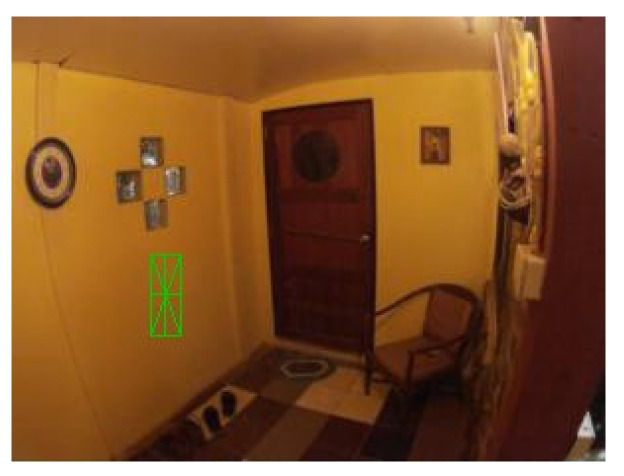
“Star of thickness 1 surrounded by a rectangle”.

The shape of the “star of thickness 1 surrounded by a rectangle” (see Figure 10) was an attempt to see if the combination of an empty rectangle and a star might lead to good results. However, this shape had the worst results among all tested shapes, and it was excluded from further testing due to its complicated shape.

**Figure 11:**
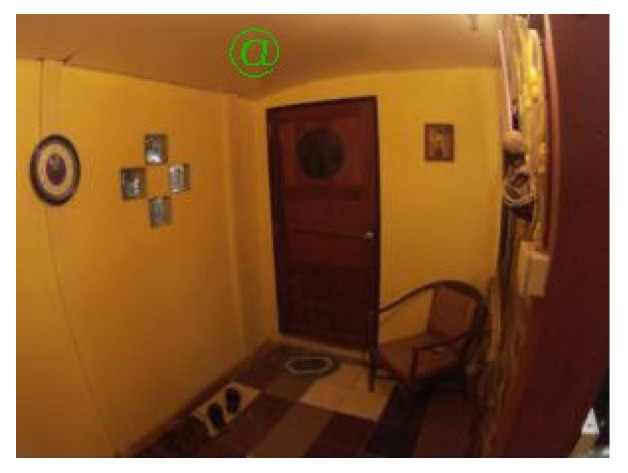
“@ symbol”.

The shape of the “@ symbol” (see Figure 11) usually achieved one of the best IoU values. Nonetheless, as it did not meet any defined criteria and had a high combined count of false positive and false negative cases, it was also rejected from another testing.

The following Figure 4, Figure 5, Figure 6, Figure 7, Figure 8, Figure 9, Figure 10 and Figure 11 show one of the testing scenes with artificially generated markers of the mentioned and evaluated shapes.

**Figure 12:**
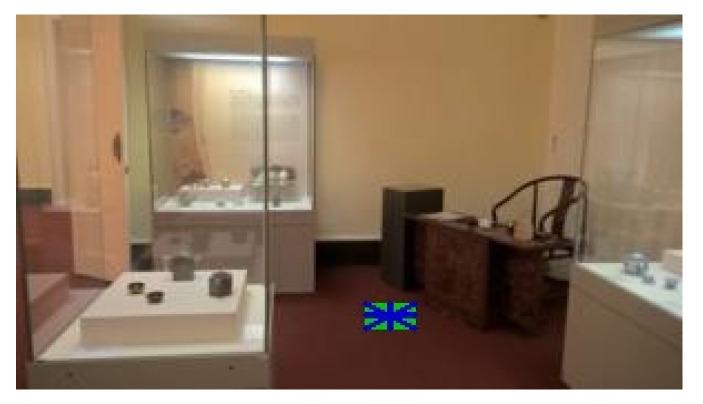
“Star of thickness 2 in a filled rectangle”.

**Figure 13:**
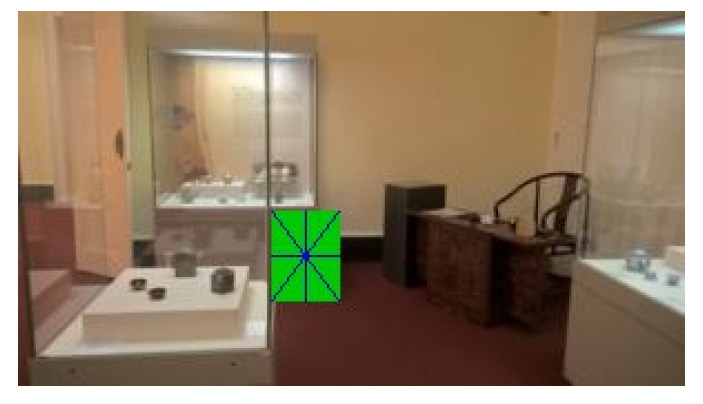
“Star of thickness 1 in a filled rectangle”.

**Figure 14:**
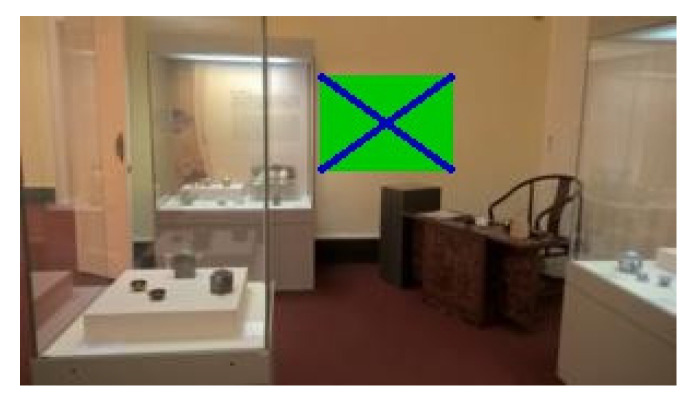
“Cross of thickness 2 in a filled rectangle”.

**Figure 15:**
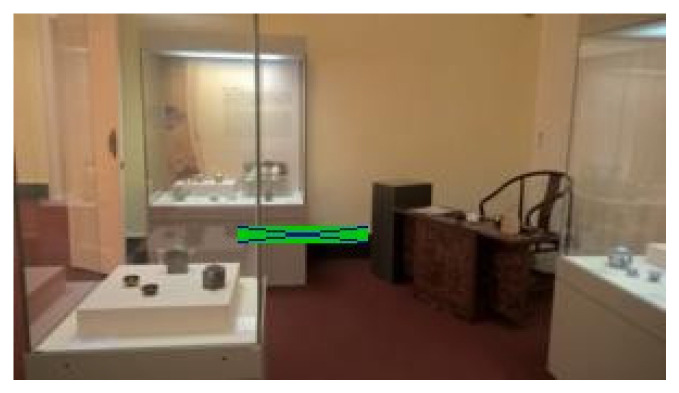
“Cross of thickness 1 in a filled rectangle”.

Four new shapes were designed based on the obtained results. These shapes were:

- star of thickness 2 in a filled rectangle (see Figure 12);
- star of thickness 1 in a filled rectangle (see Figure 13);
- cross of thickness 2 in a filled rectangle (see Figure 14);
- and cross of thickness 1 in a filled rectangle (see Figure 15).

These shapes broke the condition of having a single-colored marker because marking a star or a cross in a filled rectangle requires two colors. This step was the next direction because detecting a uniform rectangular area was prone to false positive detections. The star itself also did not achieve the expected success.

These four shapes also met three out of four defined criteria for a marker shape. They did not meet the criterion for detecting rotation in 360° resolution as it was only possible to detect the rotation in 90° or 180° resolution.

All two-colored variants gained remarkable results, usually with very low false positive counts (sometimes very close to zero) and low enough counts of false negative detections (see Table 3). That meant that these shapes were a step in the right direction, and the work was focused on revising the ideas to match all four defined criteria.

### 4.2. The “T cross” Shape

**Figure 16:**
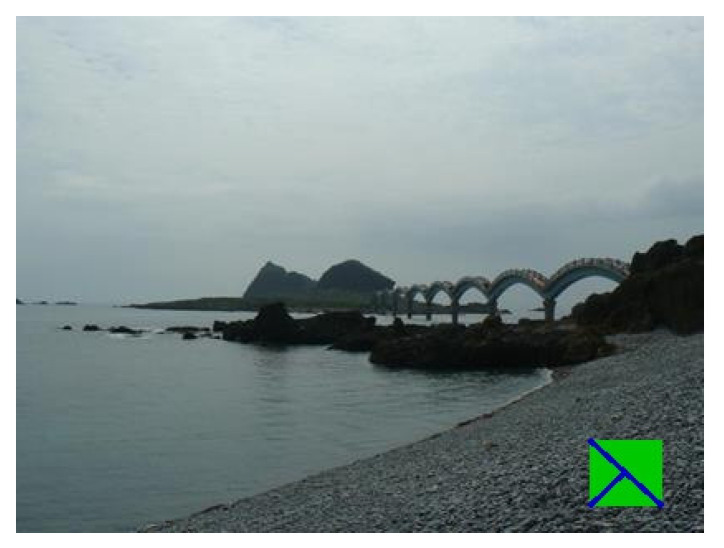
Shape “T cross” of thickness 2.

**Figure 17:**
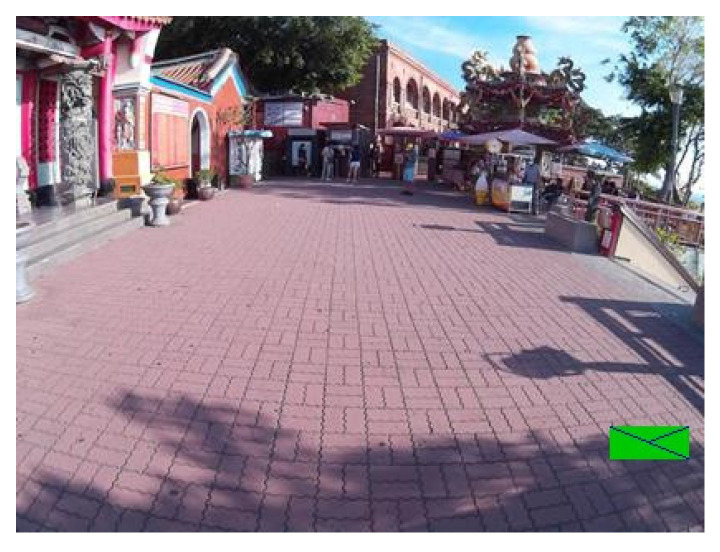
Shape “T cross” of thickness 1.

After the prior tests, it was clear that two-color marker shapes represent the right direction to follow. Both tested two-colored variants (star and cross) achieved almost identical results, and the cross is the simpler shape preferred to the star. The problem with the cross shape (and the star shape, too) is that rotation detection can only be done in the range up to 90°. When the rotation angle exceeds 90°, it is no longer possible to know which rotation occurs if the shape is square. Since this is one of the primary criteria for the shape of the marker, a new variant of the marker was designed, allowing rotation resolution in the range of up to 360°. The possible solution is a cross that lacks one part leading to one of the corners, called “T cross” (see Figure 16 and Figure 17).

The same training on the same dataset with the same number of epochs was performed to see if this shape has a similar performance. The numbers of false positives and negatives were comparable, with the IoU metric moving down by about 4–5 hundredths (see Table 4). Consequently, this shape was chosen as the final shape because it had good detection results and met all defined criteria:

- *Localization of the marker*—the shape has a center to detect, and the center is detectable from the three lines going from the center to the three corners.
- *Detection of rotation angle in 360° resolution relative to the viewer*—the shape has three lines present in a way that allows detection of described rotation.
- *Detection of the whole marker to obtain its relative size*—the shape has a filled area that can be detected.
- *Shape of the marker that supports obtaining perspective transformation*—the shape has a filled area that can be used for this purpose.

The final test on artificial markers was performed for the enlarged dataset of 10,797 images, divided into 7560 training, 2158 validation, and 1079 testing datasets. The test proved that expanding the dataset positively affects the resulting detections (see Table 5).

A combined test with marker shape “T cross” with the random thickness of one or two was also performed. It was supposed to simulate more realistic situations, and it is important that the results were comparable with the previous ones (see Table 5).

Overall, precision was kept over 0.99, and false positive detections were almost eliminated. However, false negative detections are challenging to get below 4%. They remain a problem that will need to be further addressed. The mean IoU in some places exceeded 0.76, which means excellent detections.

### 4.3. Markers in Real Scenes

The final test was performed on real markers in natural scenes. A total of 18 markers of nine different colors were drawn, and a video for each marker was taken. Five videos were taken outside and thirteen inside. The outside environment usually presents a more significant challenge due to higher variability in lighting conditions. Those five outside videos were recorded on an ordinary summer day at around 11 am. There was a slight haze, so the sunlight was not direct. The videos were taken in 1280 × 720 resolution with 30 frames per second on an ordinary smartphone (Honor 9). A total of 550 images were captured from the videos. Generally, every 10th frame was grabbed. However, some frames did not contain any markers, and those frames were deleted. Each resulting image contained exactly one marker.

**Figure 18:**
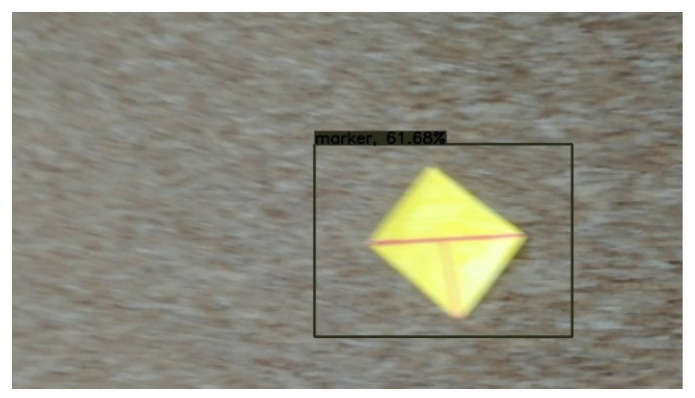
Example of a mild motion blur, the image illustrates how the “T cross” shape behaves well under motion blur as one of the lines is still almost sharp.

The fact that images were captured from videos is crucial as it presents a real-life scenario, and around half of the grabbed images suffered from mild motion blur. A small number of images have such a large amount of motion blur that blur’s length represented up to 40% of the marker’s total width. Those are challenging conditions, but as they can occur in natural scenes when camera movements are quick, it is necessary to consider them. The “T cross” shape shows one of its advantages when dealing with motion blur. The shape contains two lines that cross close to 90° angle. That means that with motion blur in any direction, at least one of the lines is still sufficiently clear (see Figure 18).

**Figure 19:**
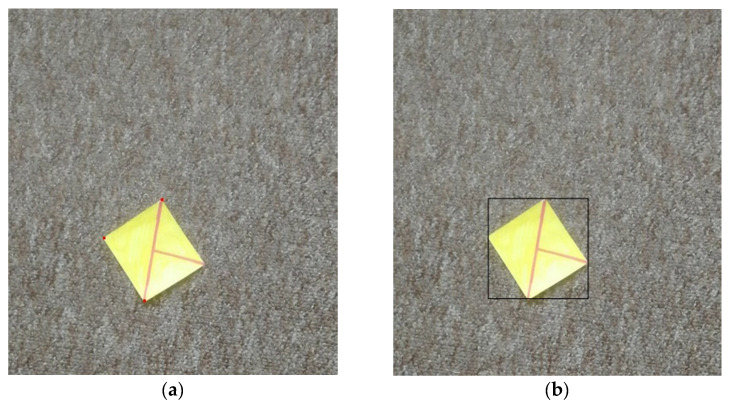
Illustration of the tagging process (**a**) three red points are placed (**b**) the points are replaced with the bounding box after the fourth point is placed.

All images were manually tagged with a ground truth bounding box. A methodology proposed by Papadopoulos et al. [82] was used for tagging. The process consists of clicking on the top, right, bottom, and right-most parts of the tagged object, in this case, the rectangular marker (see Figure 19). This method is supposed to be faster than extending prepared lines to wrap the tagged object.

The dataset was divided into 385 train images, 110 validation images, and 55 test images. The training was performed for 50 epochs, with the best results gained at epoch 37. Out of 55 images, four false negative cases and one false positive case were detected with the default detection threshold of 0.3 (see Table 6).

The final training was further tested with various detection thresholds. In general, the threshold is set to determine which bounding boxes will be considered and which will be discarded. The neural network assigns each detection a probability, which describes how sure the network is of the detection. The default value is 0.3 (30%). This threshold can be set to a lower value, which leads to a lower number of false negative detections. However, the number of false positive detections gets higher. Accordingly, the precision and recall measures are influenced. In our case, by setting the threshold to 0.25, we got three false negative detections and one false positive detection (see Table 6).

**Figure 20:**
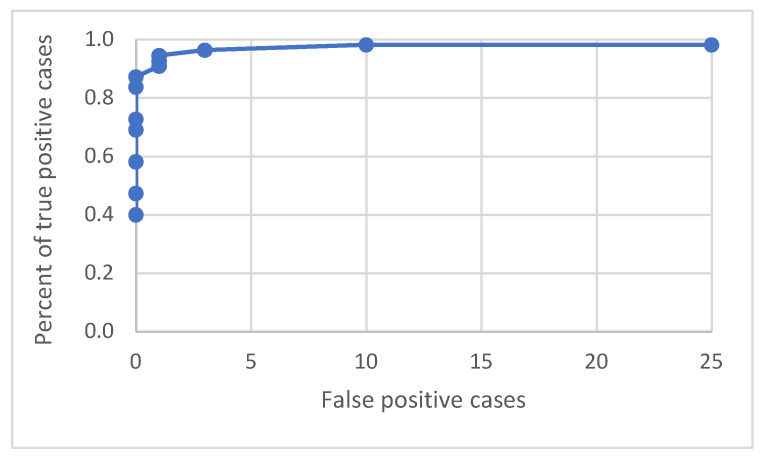
ROC curve for various detection probability thresholds in the range of 0.05 to 0.7.

An ROC curve was built to investigate the relation between true positive and false positive detections. As the following graph with the curves shows (see Figure 20), the detection process works well by having a low number of false positive and a high percentage of true positive cases.

### 4.4. Examples of Detections of Real Markers

**Figure 21:**
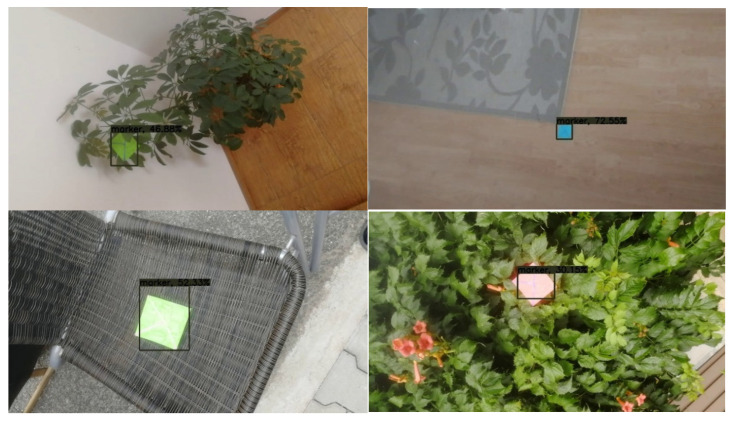
Examples of detected markers that were manually drawn. Two top images are taken in an inside environment, and two bottom images were taken outside.

The following figures show examples of different detected hand-drawn markers in real scenes. Figure 21 contains four images, each representing one detected marker in various natural scenes. Two of the selected images are in an indoor location, and the other two are in an outside environment. The figure demonstrates that the detection process works well in different scenes.

Figure 18 contains an example of a marker with a high level of motion blur. The marker is still well detected. The shape “T-cross” behaves nicely under motion blur when one line of the marker is still very clearly visible.

**Figure 22:**
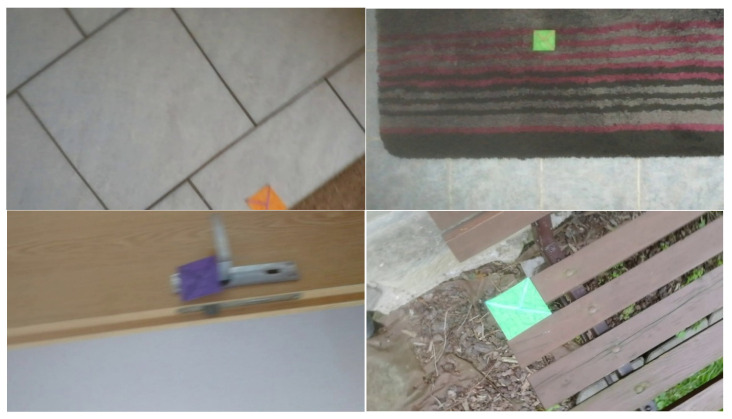
The four false negative cases with 0.3 detection probability threshold out of the 55 test images. The top two images are detected if detection probability threshold is set to 0.2.

**Figure 23:**
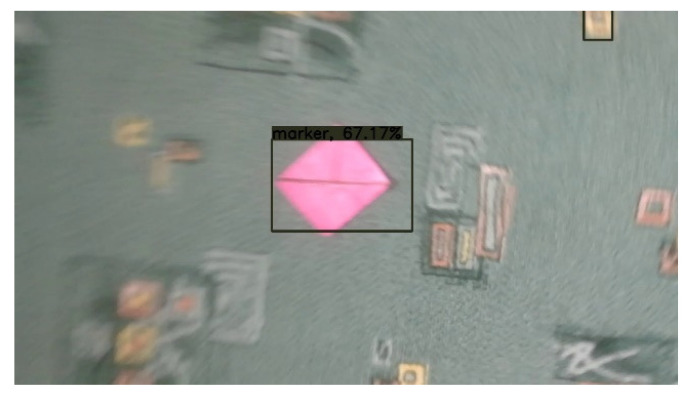
The only detected false positive case with 0.3 detection probability threshold; part of the carpet in the top right corner is falsely detected as a marker.

Figure 22 contains the only four false negative detections for the given dataset of real images. Figure 23 demonstrates an image that contains the only detected false positive area, where part of the carpet is falsely detected as a marker.

### 4.5. Summary of the Shape Design

The final marker shape is called “T cross”, and it meets all four defined criteria for a marker shape. Therefore, it can be used in augmented reality applications because it can provide all essential information. The tests on both artificial and real markers prove that the “T cross” shape can provide the specified information from the spatial scene. The tests have also shown that the shape has excellent detectability with low counts of false positive and false negative cases and a high IoU value. The implemented solution uses the YOLOv3 architecture of an artificial neural network.

### 4.6. Getting Information from the Detected Marker

After the detection process, the detected area is used to get all the described necessary information that is the whole solution’s output. At first, it is necessary to filter out the pixels that are not part of the marker. This is done by converting the image from the RGB to the HSV color model, building the histogram of hue values, and getting the hue with the largest count of pixels. Then, this obtained hue is used to filter out all other pixels with different hues outside of the specified interval around the base hue. After that, a set of classical algorithms of computer graphics follows. At first, it is morphological operation erosion to fill noise spaces in the image. After that, dilatation operation follows to get back to full marker size, which must be preserved for later use.

At this point, the image already contains well-apparent marker contours, and it is possible to obtain the lines. Before applying the Hough Lines algorithm, it is necessary to process the image with Gaussian blur and Otsu’s adaptive thresholding to get a binary image on which Canny’s detector can precisely detect the marker’s boundary lines. And then finally, the Hough Lines algorithm can be performed. It is always assured that at least ten lines are returned, but not more than 40. That is achieved by modifying the threshold parameter of the algorithm.

To sum up the process in short steps:

1) Crop the image with the detected coordinates and fixed padding.
2) Convert the image from the RGB to the HSV color model.
3) Build a histogram of hue values and get the hue with the largest count of pixels.
4) Filter out all other pixels with different hues.
5) Perform morphological operation erosion.
6) Perform morphological operation dilatation.
7) Apply Gaussian blur to the image.
8) Apply Otsu’s adaptive thresholding.
9) Apply Canny’s edge detector.
10) Apply Hough Lines algorithm.

The detected lines contain enough data to obtain all the necessary information. At first, the lines are divided into four clusters by their different slopes. Two of these clusters then contain lines of the outer quadrilateral area. They can be distinguished by containing two clusters of y-intercepts–these two groups have a much higher standard deviation of these values where the other two groups have it very close to zero. Finally, after the groups are divided, the position is calculated as an intersection of the groups of lines that form the inner “T-cross” shape.

To get the orientation, it is further necessary to decide which of the inner lines is the shorter one. After that, the orientation vector is obtained by looking at which of the points of the shorter line is closer to the center. Then the other point represents the direction for the orientation vector. And finally, to get the size, it is necessary to divide the clusters of the outer lines each into two other clusters by their y-intercepts. After that, the intersections of the four lines are representing the outer shape of the marker.

### 4.7. Examples of the Information-Obtaining Process 

**Figure 24:**
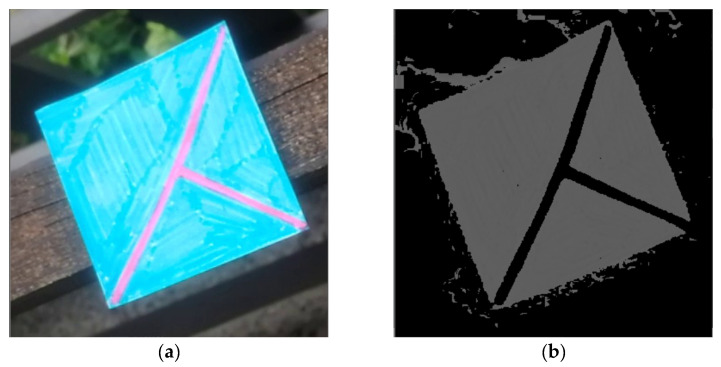
(**a**) cropped marker from the detected area of the original image. (**b**) image after filtering out pixels without the most common hue.

**Figure 25:**
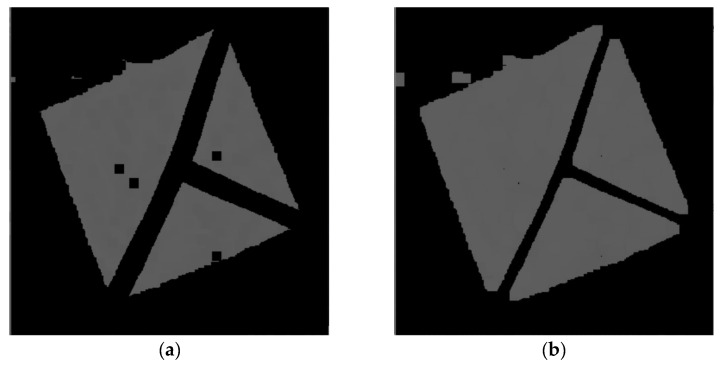
(**a**) image after performing erosion operation. (**b**) image after performing dilatation operation.

**Figure 26:**
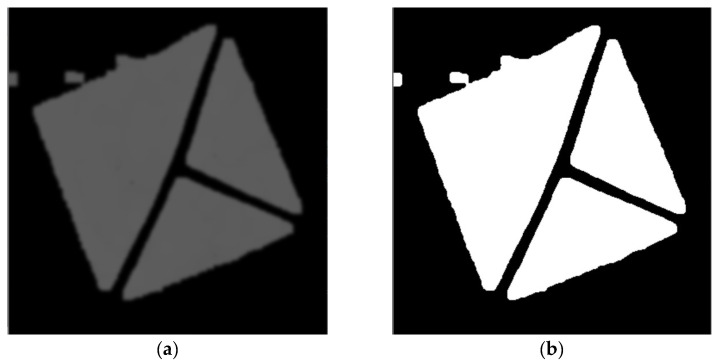
(**a**) image after applying Gaussian blur. (**b**) image after applying Otsu’s adaptive thresholding.

**Figure 27:**
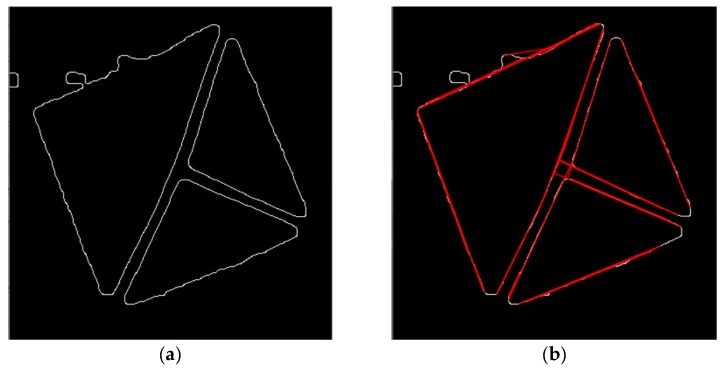
(**a**) image after applying Canny’s edge detector. (**b**) the same image with resulting lines of the Hough Lines algorithm lines being drawn to it.

**Figure 28:**
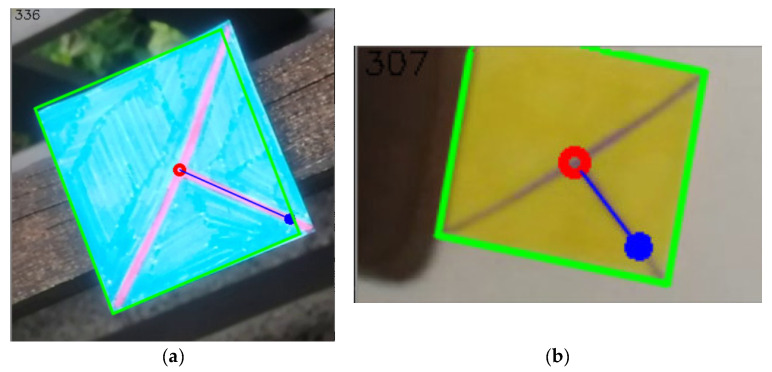
(**a**) the resulting image after getting information of the position (red circle), orientation (blue orientated line, the angle also being written in the left top corner of the image), and size (green outline). (**b**) the same set of information but for a different image.

**Figure 29:**
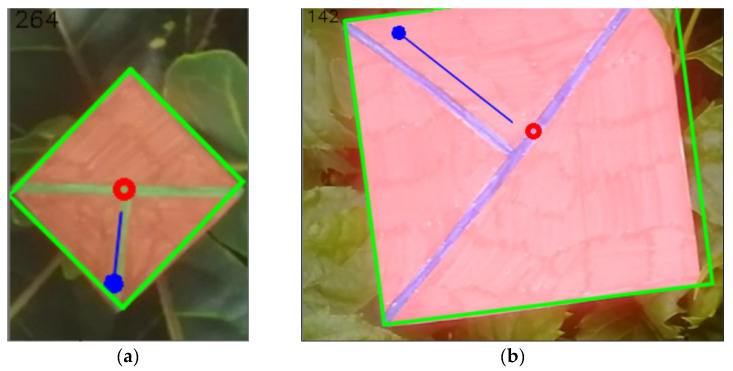
(**a**) the resulting image after getting information of the position (red circle), orientation (blue orientated line, the angle also being written in the left top corner of the image), and size (green outline). (**b**) the same set of information but for a different image.

The following Figure 24, Figure 25, Figure 26, Figure 27, Figure 28 and Figure 29 illustrate individual steps after passing the image through the designed algorithm.

## 5. Discussion

Fifteen different marker shapes were tested through all the tests. That enabled a thorough understanding of which shapes are easily detectable and have high and low counts of false positive and false negative cases and satisfactory IoU metric. Based on all these tests, the best marker shape is called “T cross”. It meets all four defined criteria for a marker shape, and at the same time, has superior results in detectability, too.

Throughout the tests, single-colored markers did not achieve good results. They also did not meet all defined criteria. Consequently, two-colored marker variants were proposed. They proved to have better results, and with the suitable design, they also met all the defined criteria.

The chosen shape had in the final test with artificial markers precision of 0.995, which means that false positive detections are almost eliminated. False negative counts remain a problem as they seem to be hard to get under 3%, which corresponds to a recall value of around 0.97. IoU is often reaching a value close to 0.8, which means excellent precision of true positive detections. This metric is important as two out of four criteria depend on it—marker size and projection transformation inevitably need the marker to be detected accurately.

The test with real markers proved that the idea is applicable in natural scenes. Obtaining more extensive datasets is very time-consuming, and so the test with only 550 images was performed. Nevertheless, even with such a number of markers, the detection results were satisfactory and worked well as proof of the concept (see Table 7).

The following processing proved that obtaining all necessary information of position, orientation, and size from the detected marker is possible.

As explained before, there are two intended ways of marker generation. The first one, which is also more emphasized, is drawing the markers by hand. It is considered one of the main advantages of the new solution. However, it is still possible to print a marker generated by a computer, which is the second option to marker generation. The algorithm for the generation is simple, and it is not necessary to employ any complicated solution from the machine learning family, e.g., GANs [83], which are suitable for the generation of new content when the required features are complicated. The main goal of the marker is to be simple, and therefore, only a simple and well-tested algorithm can provide predictable results.

The detection system has a real-time performance. The neural network processes a single image in around 44 milliseconds on the NVIDIA GeForce GTX 1060 Mobile graphics card, which allows the processing of approximately 23 frames per second. The follow-up algorithm that obtains the information from the detected marker takes, on average, 11 milliseconds. Totally, that adds up to 55 ms (18 frames per second). It must be noted that other systems can provide better time performance.

The cost of the solution is always the same for any image size because the YOLOv3 architecture processes input images that must be resized to the defined dimensions. Therefore, the pass through the network takes the same time for any photo. Furthermore, the follow-up algorithm that obtains information from the detected marker works with cropped images, which are processed by standard algorithms of computer graphics. Namely, they are morphological operations of erosion and dilatation, Gaussian blur, Otsu’s adaptive thresholding, Canny’s edge detector, and Hough Lines algorithm. These are well-established algorithms that provide excellent performance for the given task.

The newly developed system provides several advantages compared to QR codes. Firstly, our system is focused on augmented reality applications. That means that it does not encode any complex textual information, which is unnecessary for such applications and adds a layer of possible complications. The marker of our system can also be drawn only by hand, which is nearly impossible to do with QR codes given their complicated shape that needs to be very precise for the detection to work. Since the main target applications of our system are augmented reality applications, it very important that our system allows markers of different colors. Support for other than grayscale colors is limited in the QR code system [84]. That means that our system can provide better blending with the surrounding environment. Our system also works well with motion blur as opposed to QR codes which need a preprocessing step to mitigate possible motion blur effects.

QR code detection and decoding were tested on the same device to get comparable speed results. OpenCV implementation of the detector and decoder was used [85]. The average speed of the detection for an image containing a single QR code was 50 milliseconds allowing 20 frames per second. That is a little faster than our system, but it is very similar.

The introduced solution is applicable in a wide range of potential applications. The general target area is augmented applications, and the main goal was to develop a method that focuses on localization for such applications. Therefore, the solution is usable in any scenario which requires localization in a spatial scene. So generally, it can be used in any augmented reality application that requires working with the environment and requires the placement of virtual objects into a natural scene. For instance, if someone wants to test a furniture placement in an empty room before purchasing it, our system can provide all the necessary information for such placement. Similarly, any other application like that one can use our solution to get the necessary information from the spatial scene.

## 6. Conclusions

The contribution introduces a simple enough marker shape that can be drawn by hand. The designed shape meets all four substantial defined criteria. The main advantage of the new marker shape is the fact that it is designed explicitly for localization which means that it is better suited for augmented reality applications than previous systems. The accuracy of the detected position is high, which ensures that the target applications can rely on obtaining precise data.

Using a neural network for the implementation is both advantageous and disadvantageous. This approach requires an extensive dataset for the training and creating it might be time-consuming. However, if the dataset is large, rich, and properly designed, it solves motion blur issues, a long distance from the marker, illumination changes, and strong shadows. This approach’s main drawback is the requirement of powerful hardware and possibly a long time for neural network training. Nonetheless, testing on a smaller dataset of real manually drawn markers proved that the design is usable in typical real-world scenes.

The research further focused on obtaining information from the marker as defined by the criteria. An algorithm that uses a set of classical algorithms of computer graphics and which culminates with the Hough Lines algorithm was developed. The testing has shown that the algorithm can provide the described information and that the “T-cross” shape is suitable for such scenarios.

Future research can focus on developing a better single-pipeline solution using a custom neural network that would detect the marker and return the required information in a single pass, leading to faster processing.

## Figures and Tables

**Table 1:** Chronological summary of the investigated marker systems.

Authors [Reference]	Marker Name/Title	Year	Citations	Base Shape	Colors
-	IGD	1990s	-	square	black
-	Maxicode	1992	-	square	black
-	QR code	1994	-	square	black
-	HOM	1994	-	square	black
Rekimoto	Matrix	1998	459	square	black
Kato	ARToolKit	1999	3469	square	black
Cho and Neumann	-	2001	75	circle	colors
-	SCR	2001	-	square	black
López de Ipiña et al.	TRIPtag	2002	298	circle	black
Naimark and Foxlin	-	2002	387	circle	black
Claus and Fitzgibbon	-	2004, 2005	84 + 59	square	black
Fiala	ARTag	2005	1060	square	black
Dell’Acqua et al.	-	2005	10	square	colors
Kaltenbrunner and Bencina	ReacTIVision	2007	572	other	black
Sattar et al.	Fourier Tag	2007	66	circle	grayscale
Flohr and Fischer	BinARyID	2007	28	square	black
Schweiger et al.	SIFTtag, SURFtag	2009	31	square	grayscale
Atcheson et al.	CALTag	2010	130	square	black
Xu and Dudek	Fourier Tag	2011	31	circle	grayscale
Olson	AprilTag	2011	1085	square	black
Bergamasco et al.	RUNE-Tag	2011	117	circle	black
Reuter et al.	BlurTags	2012	8	square	black
Li et al.	CoP-Tag	2012	6	square	black
Calvet et al.	C^2^Tag	2012	23	circle	black
Bergamasco et al.	Pi-Tag	2013	60	square	black
Liu et al.	-	2013	11	triangle	colors
Toyoura et al.	Mono-spectrum marker	2013	17	square	colors, black
Klokmose et al.	BullsEye	2014	13	circle	black
Garrido-Jurado et al.	ArUco	2014, 2016	1389 + 329	square	black
Prasad et al.	-	2015	12	circle	black
Wang and Olson	AprilTag 2	2016	340	square	black
Calvet et al.	CCTag	2016	43	circle	black
DeGol et al.	ChromaTag	2017	42	square	colors, black
Wang et al.	HArCo	2018	6	square	black
Susan et al.	-	2018	1	square	black
Benligiray et al.	STag	2019	9	square	black
Krogius et al.	April Tag 3	2019	26	square or circle	black
Yu et al.	TopoTag	2020	7	various	black

**Table 2:** The comparison of eight tested initial marker shapes of the first iteration.

Marker Shape	Mean IoU	False Positive Cases	False Negative Cases	Percent of False Negative Cases	Precision	Recall
Filled rectangle	0.665	15	16	5.4%	0.949	0.946
Empty rectangle	0.666	12	16	5.4%	0.959	0.946
Filled triangle	0.701	8	8	2.7%	0.973	0.973
Empty triangle	0.679	6	13	4.4%	0.979	0.956
Star of thickness 2	0.695	3	9	3.0%	0.990	0.970
Star of thickness 1	0.654	13	19	6.4%	0.955	0.936
Star of thickness 1 surrounded by a rectangle	0.614	19	21	7.1%	0.936	0.929
@ symbol	0.699	10	19	6.4%	0.965	0.936

**Table 3:** The table contains the comparison of the results of the four two-colored marker shapes.

Marker Shape	Mean IoU	False Positive Cases	False Negative Cases	Percent of False Negative Cases	Precision	Recall
Star of thickness 2 in a filled rectangle	0.755	2	9	1.9%	0.996	0.981
Star of thickness 1 in a filled rectangle	0.764	7	11	2.3%	0.985	0.977
Cross of thickness 2 in a filled rectangle	0.764	1	9	1.9%	0.998	0.981
Cross of thickness 1 in a filled rectangle	0.764	3	5	1.0%	0.994	0.990

**Table 4:** The testing of the “T cross” marker shape with uniform colors was carried out for 21 epochs with the interior images dataset.

Marker Shape	Mean IoU	False Positive Cases	False Negative Cases	Percent of False Negative Cases	Precision	Recall
“T cross” of thickness 2	0.689	6	10	3.9%	0.997	0.962
“T cross” of thickness 1	0.664	5	13	5.0%	0.980	0.950

**Table 5:** Testing the “T cross” marker shape that was performed with a combined images dataset of 10,797 images with a width of 416 pixels each.

Marker Shape	Mean IoU	False Positive Cases	False Negative Cases	Percent of False Negative Cases	Precision	Recall
“T cross” of thickness 2	0.780	5	33	3.1%	0.995	0.969
“T cross” of thickness 1	0.743	10	62	5.8%	0.990	0.943
“T cross” of random thickness	0.762	5	40	3.8%	0.995	0.963

**Table 6:** Testing the “T cross” marker shape with real hand-drawn markers in natural scenes on a dataset of 550 images was carried out with various detection probability thresholds in the range of 0.05 to 0.7.

Marker Shape	Detection Threshold	Mean IoU	True Positive Cases	False Positive Cases	False Negative Cases	Percent of False Negative Cases	Precision	Recall
T cross	0.05	0.710	54	127	1	1.8%	0.298	0.982
	0.10	0.710	54	25	1	1.8%	0.684	0.982
	0.15	0.707	54	10	1	1.8%	0.844	0.982
	0.20	0.692	53	3	2	3.6%	0.946	0.964
	0.25	0.685	52	1	3	5.5%	0.981	0.945
	0.30	0.673	51	1	4	7.3%	0.981	0.927
	0.35	0.659	50	1	5	9.1%	0.980	0.909
	0.40	0.628	48	0	7	12.7%	1.000	0.873
	0.45	0.600	46	0	9	16.4%	1.000	0.836
	0.50	0.524	40	0	15	27.3%	1.000	0.727
	0.55	0.501	38	0	17	30.9%	1.000	0.691
	0.60	0.416	32	0	23	41.8%	1.000	0.582
	0.65	0.332	26	0	29	52.7%	1.000	0.473
	0.70	0.280	22	0	33	60.0%	1.000	0.400

**Table 7:** Final comparison of the results between artificial and real markers.

Train Set	Mean IoU	False Positive Cases	False Negative Cases	Percent of False Negative Cases	Precision	Recall
Artificial (10,797 images)	0.762	5	40	3.8%	0.995	0.963
Real (550 images)	0.673	1	4	7.3%	0.981	0.927

## Data Availability

Software—https://github.com/milankostak/Marker-detection-NN/tree/v1.0 (accessed on 8 August 2021). Source repository—https://github.com/wizyoung/YOLOv3_TensorFlow (available under MIT license, accessed on 30 April 2020). Dataset of the real images—https://files.milan-kostak.cz/sensors-markers-dataset.zip (accessed on 30 April 2020).
